# Associations between perceived occupational stressors and symptoms severity of depression, anxiety and stress among academic faculty: First cross-sectional study from Qatar

**DOI:** 10.1186/s40359-024-01801-x

**Published:** 2024-05-28

**Authors:** Dalal Hammoudi Halat, Manar E. Abdel-Rahman, Ghadir Fakhri Al-Jayyousi, Ahmed Malki

**Affiliations:** 1 Academic Quality Department, QU Health, Qatar University, P.O. Box 2713, Doha, Qatar; 2 Department of Public Health, College of Health Sciences, QU Health, Qatar University, Doha, Qatar

**Keywords:** Mental health, Faculty, Faculty stress index, Job-related stress

## Abstract

**Background:**

Mental health concerns among university faculty are on the rise, with reports of anxiety, depression, and occupational stress, impacting the higher education community. In Qatar, an assessment of faculty mental health has not been previously realized. The objectives of the current study were twofold: Firstly, to evaluate the extent of perceived occupational stress, depression, anxiety, and stress, and secondly, to assess the association among these mental health parameters.

**Methods:**

A cross-sectional study was conducted among faculty using an online, self-administered, anonymous, voluntary survey. All faculty were included by sending the survey to their institutional emails. In addition to faculty demographics and general health status, the survey measured perceived stress due to academic job roles using the Faculty Stress Index (FSI) with its five distinct domains, and assessed faculty mental health using the Depression, Anxiety, and Stress Scale-21 items (DASS-21). Modified Poisson regression with robust variance was used to assess how FSI influences levels of depression, anxiety, and stress.

**Results:**

A total of 112 faculty responded to the survey. The highest faculty self-perceptions of mental health conditions were for anxiety (63% at least moderate), followed by depression (30% at least moderate), and least for stress (26% at least moderate). The overall mean FSI score was 48.8 ± 29.4; time constraint and rewards and recognition domains scored highest (18.5 ± 11.4 and 13.3 ± 9.3 respectively) while the departmental influence domain scored least (4.8 ± 4.4). Increased risk of at least moderate levels of self-perceived depression and stress were significantly associated with higher FSI score (p˂0.001). Increased risk of at least moderate levels of depression were less likely among faculty aged 50 years and above (*p* = 0.034), while increased risk of at least moderate levels of anxiety were more likely among faculty from humanities colleges (*p* = 0.027).

**Conclusions:**

This is the first investigation of university faculty mental health in Qatar, indicating multifactorial perceived occupational stress, associated with higher perceived severity of mental health conditions. These baseline results establish links between specific occupational stressors for faculty and their mental well-being. As such, assessment of mental health conditions, controlling occupational stress, and developing tailored mental health interventions for faculty, are strategic to implement and foster well-being of academics. Further research into mental health of faculty and designing effective interventions that consider their specific stressors and associated factors are warranted.

**Supplementary Information:**

The online version contains supplementary material available at 10.1186/s40359-024-01801-x.

## Introduction

The World Health Organization (WHO) defines mental health as a state of mental well-being that enables individuals to cope with life stressors, realize their own potential, learn and work efficiently, and contribute to their community and to the socio-economic development [1]. However, achieving such level of mental well-being remains a persistent struggle, with over one billion individuals globally suffering the toll of a mental or an addictive disorder [2]. Recent estimates of mental health burden point out to over 400 million disability-adjusted life years (DALYs) attributable to mental disorders, accounting for 16% of global DALYs, and an associated economic encumbrance that amounts to about 5 trillion USD [3].

Among mental disorders, depression and anxiety appear to be the most disabling conditions, and both are ranked among the top 25 leading causes of disease burden worldwide [4]. A debilitating disease characterized by depressed mood, reduced interests, and compromised cognitive function, depression affects about 6% of the adult population worldwide [5], and is the second leading contributor to chronic disease burden [6]. On the other hand, anxiety constitutes the largest plethora of mental disorders in most Western societies and persists as a leading cause of disability, with persistent fear, avoidance of perceived threats, and possibly panic attacks [7]. The global prevalence of anxiety is around 3.6% [8], and is higher in developing countries [9]. Apart from depression and anxiety, mental stress is a common and collective aspect of human existence. Evidence indicates that around two-thirds of the general population have encountered mental stress in the last two weeks, with nearly half of them describing it as “moderate or high” [10]. Stress has been pondered since ancient times, and it lingers as a product of the rapid, interconnected, and technologically advanced society of the 21st century [11], resulting in physical and mental health issues affecting individuals’ overall well-being [12]. In the workplace, occupational stress is a major issue precipitated by various job demands and experiences with short- and long-term implications [13]. Defined as “harmful physical and emotional responses that occur when requirements of the job do not match the capabilities, resources, and needs of the worker” [14], occupational stress affects a minimum of one-third of employees in various sectors, and is linked to several other disorders including insomnia, cardiovascular diseases, diabetes, and depression [15].

Across academia, mental health concerns have been documented at all levels, from undergraduate to graduate students, through junior and senior faculty [16], and calls for conversation over this issue have been on the rise [17]. For university faculty, the typically heavy workload, often tied to internal or external deadlines, competition for research resources, and uncertain job opportunities, can negatively affect mental well-being. Poor management practices, along with inadequate recognition and rewards, might further exacerbate faculty mental health status [18]. Several other factors contribute to escalate this so-called “invisible crisis” of mental health in academia [19, 20]. Among these are challenges with maintaining work-life balance, navigating interactions with students, changes in higher education and research structures, and the recent, unparalleled transformations in academia due to the lasting impact of the COVID-19 pandemic, all of which seem to influence the mental well-being of faculty [21]. As such, the constant pressured to meet various demands of student interaction [22], teaching [23], promotion [24] and other tasks exerts its toll on faculty well-being, affecting their mental health [25].

Described as being prevalent but often widely ignored [18], mental health concerns among faculty usually occur in silence. Faculty tend to conceal their mental health problems from others due to fear of anticipated stigma, consequences on their careers, and confidentiality. The stereotype of high performance and prosperous achievements, usually nurtured in faculty during their early training and education, is greatly challenged during the career span. The demands of academia consistently confront individuals with shortcomings, promote perfectionism and competitiveness, and drive high expectations. This, in turn, perpetuates to faculty the belief that mental illnesses are inherent weaknesses, and that seeking help is a barrier to academic success, easily setting the stage for symptoms of anxiety, depression, and stress among this population [26]. In a survey of faculty in 2022 in the US, more than 80% of the respondents reported lifetime history of mental-health difficulties, and nearly half reported a diagnosed mental disorder [27]. More specifically, and in a recent investigation of faculty from 10 universities, 5.5% had increased symptoms of depression, 11.5% had increased symptoms of anxiety, and 23.4% had moderate to high stress levels [28], highlighting the need for investigations and remedial actions directed towards faculty well-being.

In terms of occupational stress, and despite the teaching profession previously viewed as a low stress occupation and faculty being resented for tenure, light workloads, and flexibility [29], faculty experience higher than normal levels of stress and ranked as second employment category in terms of worse-than-average psychological well-being scores [30]. The influence of job demands and the effects of the academic environment on mental health of faculty has been previously described [31–33], and the Faculty Stress Index (FSI), initially developed by Gmelch, Wilke, and Lovrich [34] is a reasonably explored tool in this regard. Through an investigation of the multidimensionality of faculty pressures and a consideration of uniqueness of the academic career, the FSI assembles a spectrum of roles that faculty undertake as teacher, researcher, adviser, university citizen, and departmental colleague. To capture these different responsibilities, the FSI measures five domains of perceived faculty stress: (i) reward and recognition domain, which pertains to rewarding external expectations for community and university services; (ii) time constraints domain, which confronts the number of tasks faculty members usually incorporate within their professional lives, including general duties such as paperwork, meetings, phone and visitor interruptions, and sufficient time for professional development, teaching, and services; (iii) departmental influence domain, which focuses on the extent to which faculty perceive their department as controlling over their work or the level of autonomy they have within their departmental facets, such as resolving differences, knowing evaluative criteria, and influencing decisions at departmental/ institutional levels; (iv) professional identity domain, which refers to reputation as a scholar and capability of setting and achieving professional goals, and is established on the basis of publications, presentations at professional meetings, and acquiring research grants; and (v) student interaction domain, which considers classroom instruction, course preparation, test administration, and advising [33, 34]. Moreover, the FSI has been recently validated, and showed good internal consistency and reliability as an instrument useful to measure stress among faculty members [35].

Qatar University (QU) is the country’s largest national institution of higher education and continues to serve as Qatar’s primary university. Nowadays, QU has become a beacon of academic and research excellence in the Gulf region and internationally. The university is committed to providing high-quality education in areas of national priority, while aligning its programs with established international standards and best practices. QU hosts eleven colleges and offers a range of over 100 academic programs. In 2022, QU was recognized as a Healthy University by the World Health Organization (WHO). The concept of the health-promoting university is powerful, whereby it means integrating health into the culture, processes, environment, and policies of the institution. In addition, it means understanding and dealing with health within a framework that blends factors as choice and participation with goals for equity, sustainability, and health-conducive living, working and learning environments [36].

To our knowledge, a focused investigation of the mental health of faculty has not been realized previously at QU nor in Qatar, and a gap in literature exists in this regard at a national level. The objectives of the current study were twofold: Firstly, to evaluate the extent of perceived occupational stress, depression, anxiety, and stress among QU faculty, and secondly, to assess the association among these mental health parameters.

## Methods

### Study design

This study is part of a larger project aimed towards assessments of various aspects of mental health, well-being, and social determinants in a sample of faculty at QU. For this part of the project, a descriptive, cross-sectional, anonymous survey was conducted among QU faculty to assess their mental health aspects and investigate associated factors. The survey was electronic and self-administered, and faculty were asked to voluntarily complete it online. The conduct and reporting of this study follow the statement of the Strengthening the Reporting of Observational Studies in Epidemiology (STROBE) guidelines [37].

### Participants

All QU faculty members, across all nine colleges, including faculty with different ranks (professors, associate professors, assistant professors, lecturers, teaching assistants, as well as part-time faculty) were invited to fill the survey. At the time of development of the study protocol, the number of faculty at QU was reported at 1355. QU staff who were non-academics, like support staff and administrators without a faculty contract were excluded. Also excluded were faculty who did not agree on providing their consent to participate. To collect responses, a non-probability based, convenience sampling method was used.

### Data collection

Data were collected anonymously via an online survey sent to QU faculty members using their institutional email. The survey was prepared using Microsoft Forms application housed within the website of QU, and password-protected so that only the researchers can edit the questions and view responses. With support from the QU broadcasting team, a bilingual email announcement was sent to all faculty, whereby the study scope, research objectives and targeted participants were briefly described. The announcement was received only by QU faculty through their institutional emails. Participants were informed that their participation is voluntary and entails no risks nor benefits, and were ensured of anonymity and confidentiality of their responses. Completion of the survey till its end and submission of a response were considered as informed consent to participate. The email included a link and a QR code for participants to access each of the Arabic and English surveys.

### Survey instrument and study variables

The survey instrument was developed by the authors in both Arabic and English to capture responses of all faculty, whereby some, particularly in the College of Law and the College of Sharia and Islamic Studies, were not English-speakers. Before the survey was launched, and for each of the Arabic and English versions, piloting was done with 5 faculty members who were invited to fill the survey and report any comments, feedback, or vague questions to the research team, for content validation of the survey questions. The piloting responses were not included in the analysis, but were used to implement amendments on the survey for content and clarity. The final survey instrument consisted of four sections. In the first section, faculty were asked about demographic data like age, gender, nationality and marital status, as well as data about their current academic position including their affiliated college, their highest academic degree, years of employment at QU, employment type (full-time or part-time), and whether they held any administrative tasks as academic administrators.

The second section of the survey was used to collect variables about general health and lifestyle habits of participants, including hours of sleep, physical activity, smoking (including cigarettes, vape and/or hookah). Also, participants were asked whether they have any medical diagnosis among hypertension, heart disease, diabetes, dyslipidemia, chronic kidney disease, chronic lung disease, or cancer. Another question inquired about participants’ diagnosis of a mental health disorder like anxiety, depression, schizophrenia, panic attacks, bipolar disorder, eating disorders, or others.

The third section of the survey included the FSI to evaluate perceived stress in academic settings considering different faculty roles, as previously described [31, 34, 35]. Briefly, 28 statements were used to measure the five domains of this instrument: reward and recognition domain was measured by 7 items, time constraint by 10, departmental influence by 3, professional identity by 3, and student interaction by 5. Participants were asked to rate the statements on an increasing score ranging from Not Applicable Pressure (0) to Very Slight Pressure (1), Slight Pressure (2), Moderate Pressure (3), Some Pressure (4), and Excessive Pressure (5). The higher the score on the sum of all the items, the higher would be the perceived faculty stress. Also, the higher the score on summation of statements for a particular domain, the higher would be faculty stress related to that domain. Bilingual members of the research team who were native Arabic speakers translated the English version of the FSI into Arabic.

The fourth section of the survey was intended to assess mental health of participants using the standardized Depression, Anxiety, Stress Scale-21 items (DASS-21) [38] and its Arabic translation [39]. This instrument included a set of three self-reported scales which provide independent measures of depression, stress, and anxiety, with recommended severity thresholds, and has been validated in the Arabic language according to previous studies [40, 41]. Each of the three DASS-21 scales contains seven items, divided into subscales with similar content. The depression scale assesses dysphoria, hopelessness, the devaluation of life, self-deprecation, lack of interest/involvement, anhedonia, and inertia. The anxiety scale assesses autonomic arousal, skeletal muscle effects, situational anxiety, and the subjective experience of anxious affect. The stress scale is sensitive to levels of chronic nonspecific arousal. It assesses difficulty relaxing, nervous arousal, and being easily upset/agitated, irritable/over-reactive, and impatient. Each of the 21 items comprises a statement and four short-response options to reflect severity, scored from zero to 3. Participants were asked to read each statement and choose how much the statement applied to them over the past week, on a rating scale of: zero (Never - Did not apply to me at all); 1 (Sometimes - Applied to me to some degree or some of the time); 2 (Often - Applied to me to a considerable degree or a good part of time); or 3 (Always - Applied to me very much or most of the time). Scores for depression, anxiety and stress were calculated by summing up the scores for the relevant items (statements 3, 5, 10, 13, 16, 17, 21 for depression; statements 1, 6, 8, 11, 12, 14, 18 for stress; statements 2, 4, 7, 9, 15, 19, 20 for anxiety). As previously reported, the Cronbach’s alpha for the DASS-21 subscales were 0.886 for depression, 0.84 for anxiety, and 0.871 for stress, indicating good internal consistency [42]. The severity of the three DASS-21 scales were computed and expressed as normal, mild, moderate, severe, and extremely severe. An English version of the survey used in this study is included in Supplementary file 1.

### Statistical analysis

Data was analyzed using means and standard deviations for continuous variables and percentages for categorical variables. Bivariate analysis was conducted using Chi-square tests, Fisher exact tests, and independent two-sample t-tests. Modified Poisson regression with robust variance were used to examine both crude and adjusted association between levels of depression, anxiety, stress and FSI. Levels of depression, anxiety, and stress were further classified into two groups—Normal/Mild or Moderate (and above), as per standard cutoff points indicated in Table 2. Crude and adjusted prevalence ratios with 95% confidence intervals were reported. Power calculations were performed at the conclusion of the study, considering the correlation coefficients among Depression, Anxiety, Stress, and FSI. Owing to the scarcity of similar studies, power was estimated assuming moderate correlation coefficients of 0.3 and 0.5. With these coefficients, an effect size of 0.15, a Type I error rate of 0.05, and a total sample size of 112, the calculated power of the study ranged from 45 to 66%. Stata version 18.0 was used for all analyses.

## Results

A total of 112 QU faculty participated in this research study (Table 1). The majority of the participants were Non-Qatari, PhD holders, full-time employees, males (54%), and aged less than 50 years old (64%). Nearly 45% of the participants had been at QU for more than five years, 43% were involved in administrative roles, and 55% were from non-health colleges.

**Table 1:** Participants’ demographic characteristics (*n* = 112)

	*n* (%)
**Age in years, mean(SD)**	44.2 (9.2)
**Age in years**	
<30	6 (5.4)
30–39	30 (26.8)
40–49	36 (32.1)
50+	39 (34.8)
Missing	1 (0.9)
**Gender**	
Female	52 (46.4)
Male	60 (53.6)
**Nationality**	
Non-Qatari	95 (84.8)
Qatari	17 (15.2)
**College**	
Arts and Science	25 (22.3)
Business and Economics	6 (5.4)
Dental Medicine	1 (0.9)
Education	5 (4.5)
Engineering	7 (6.2)
Health Sciences	9 (8.0)
Law	6 (5.4)
Medicine	7 (6.2)
Nursing	6 (5.4)
Pharmacy	3 (2.7)
Sharia and Islamic studies	12 (10.7)
Other colleges or departments	25 (22.3)
**Highest academic degree**	
BSc	2 (1.8)
Master	25 (22.3)
PhD	85 (75.9)
**Rank**	
Professor	5 (4.5)
Associate Professor	11 (9.8)
Assistant Professor	15 (13.4)
Lecturer	11 (9.8)
Teaching Assistant	4 (3.6)
Academic Rank	5 (4.5)
Other	7 (6.2)
Missing	54 (48.2)
**Administrative role**	
No	64 (57.1)
Yes	48 (42.9)
**Work duration at QU in years**	
<=2	31 (27.7)
>2 to 5	31 (27.7)
5+	50 (44.6)
**Employment type**	
Full-time	103 (92.0)
Part-time	9 (8.0)
**Sleep, at least 7 h per night**	
No	55 (49.1)
Yes	57 (50.9)
**Physical activity, at least 150 min per week**	
No	58 (51.8)
Yes	54 (48.2)
**Smoking, cigarettes/ vape/ nargileh**	
No	103 (92.0)
Yes	9 (8.0)
**Medical diagnosis** ^**&**^	
No	85 (75.9)
Yes	27 (24.1)
**Mental health diagnosis** ^**&&**^	
No	97 (86.6)
Yes	15 (13.4)

^**&**^Includes hypertension, heart disease, diabetes, dyslipidemia, chronic kidney disease, chronic lung disease, or cancer

^**&&**^Includes schizophrenia, panic attacks, bipolar disorder, eating disorders, or others

In regards to their lifestyle and health status, 48.2% of the participants performed at least 150 min of physical activity per week, only half of them slept at least 7 h per night, and nearly a quarter have been diagnosed with a medical condition. About 13% of the participants reported being diagnosed with a mental health condition.

**Fig. 1:**
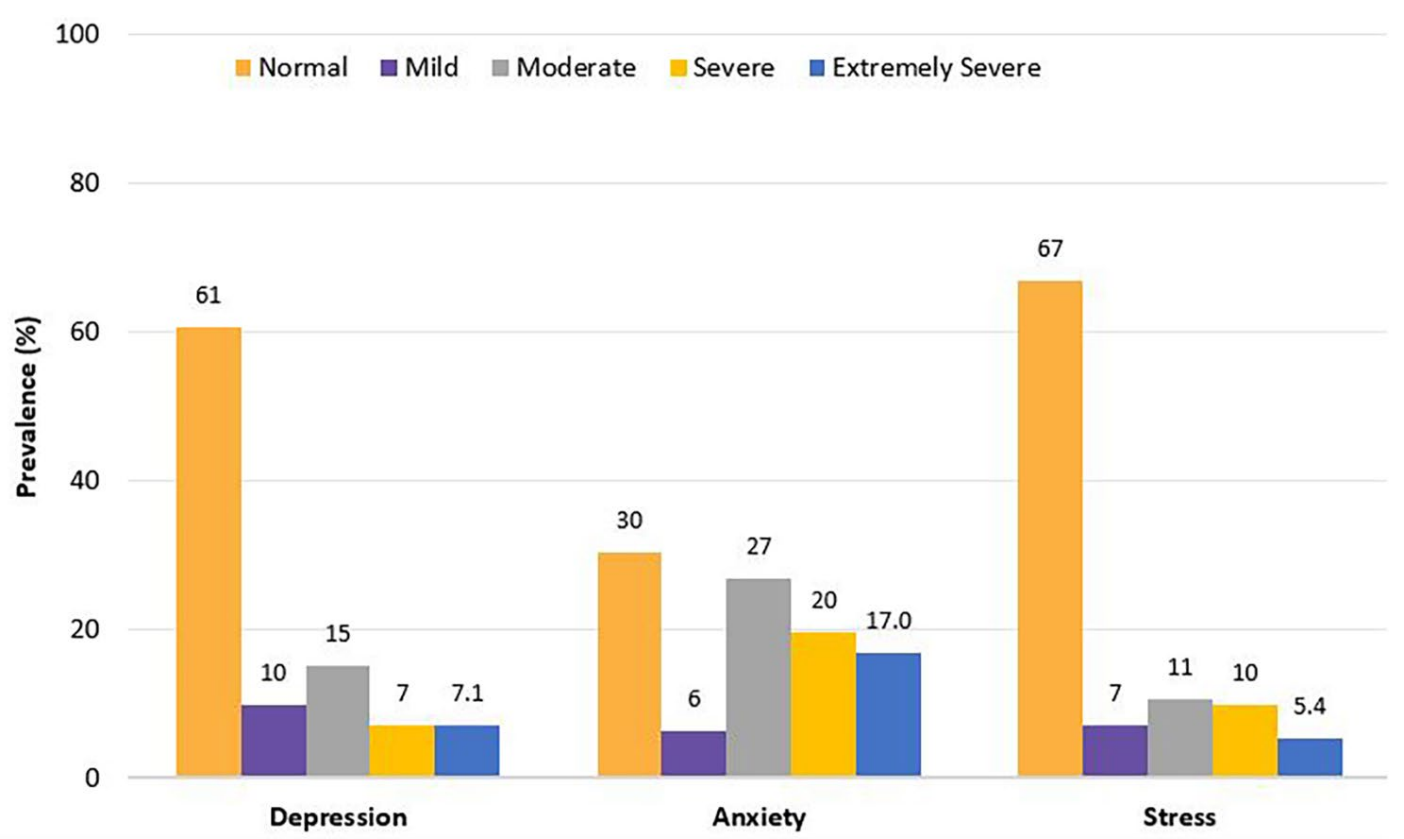
Prevalence of depression, anxiety, and stress subscales

The descriptive analyses showed that the mean scores of depression, anxiety, and stress were 9.4 ± 9.8, 11.9 ± 7.8, and 12.9 ± 10.5, respectively (Table 2). About 30% of the participants reported at least ‘moderate’ (14+) depression symptoms severity, 63.4% reported at least ‘moderate’ (10+) anxiety symptoms severity, and almost 26% of the participants perceived their stress symptoms severity as at least ‘moderate’ (19+). The prevalence of anxiety, depression and stress among participants is shown in Fig. 1.

**Table 2:** Descriptive statistics for depression, anxiety, and stress subscales (*n* = 112)

Depression (0–42), mean(SD)	9.4 (9.8)
**Depression (0–42), median [IQR]**	6.0 [13.0]
**Depression, n(%)**	
Normal/ Mild (0–13)	79 (70.5)
At least ‘moderate’ (14+)	33 (29.5)
**Anxiety (0–42), mean(SD)**	11.9 (7.8)
**Anxiety (0–42), median [IQR]**	12.0 [10.0]
**Anxiety**	
Normal/ Mild (0–19)	41 (36.6)
At least ‘moderate’ (10+)	71 (63.4)
**Stress (0–42), mean(SD)**	12.9 (10.5)
**Stress (0–42), median [IQR]**	10.0 [15.0]
**Stress, n(%)**	
Normal/ Mild (0–18)	83 (74.1)
At least ‘moderate’ (19+)	29 (25.9)

**Fig. 2:**
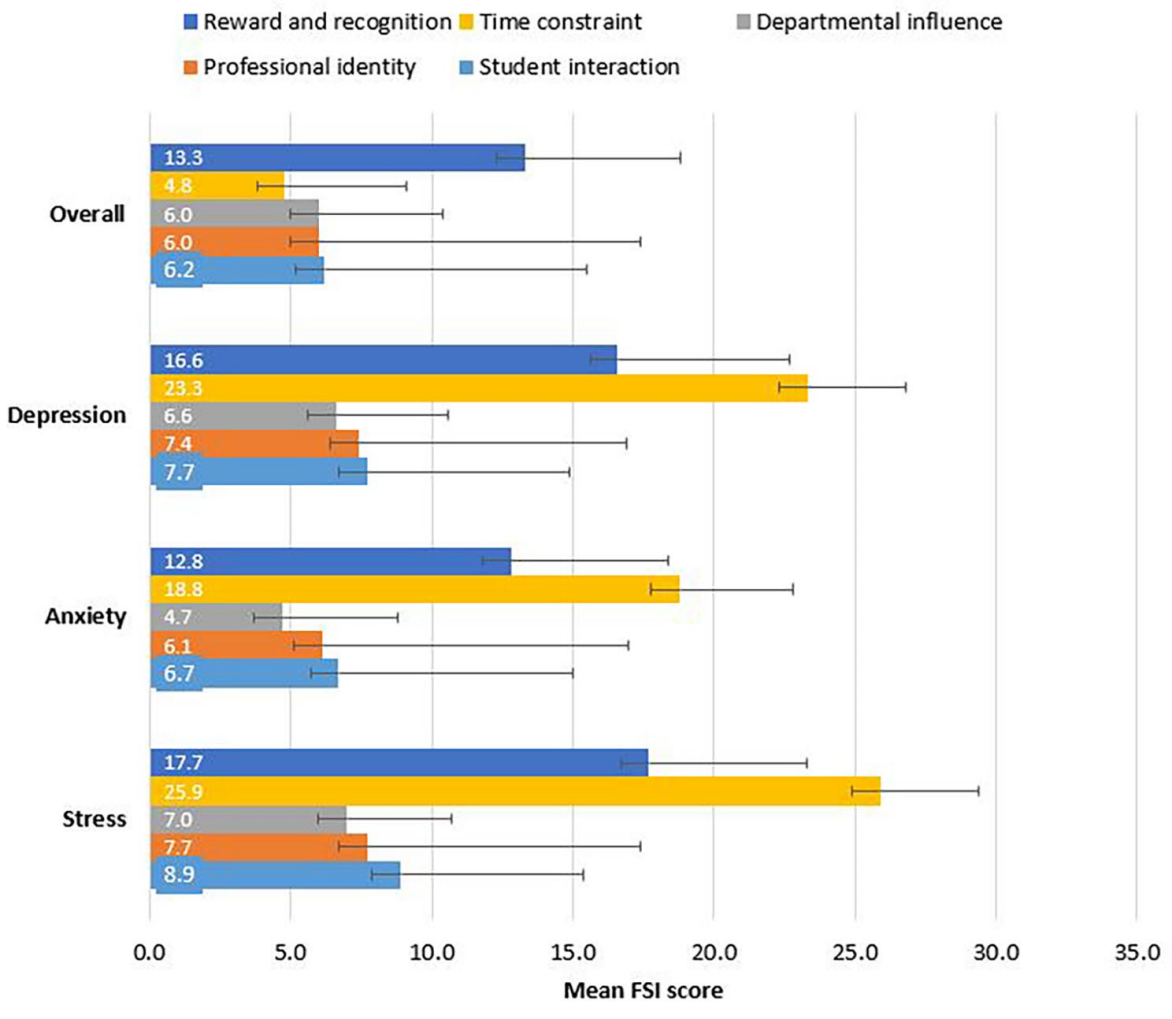
Mean FSI scores (error bars represent standard deviation)

Participants’ perception of their stress was assessed by applying the FSI represented by its five domains: reward and recognition, time constraint, departmental influence, professional identity, and student interaction (Table 3). The FSI total score mean (*M*) was 48.8. Participants reported the highest stress score under the time constraint domain (*M* = 18.5), followed by reward and recognition domain *(M* = 13.3), and student interaction (*M* = 6.2). Meanwhile, the least stress score was reported under the departmental influence domain (*M* = 4.8). The domains mean FSI scores are represented in Fig. 2.

**Table 3:** Descriptive statistics for Faculty Stress Index (FSI) score and Cronbach’s α values for this instrument

	Number of items	Total possible Score	Mean	SD	Median	IQR	Min	Max	Overall Cronbach’s α	Cronbach’s α for Arabic version	Cronbach’s α for English version
FSI total score	28	140	48.8	29.4	49.5	45.5	0	112	0.93	0.93	0.94
FSI domains scores											
Reward and recognition	7	35	13.3	9.3	12.5	15.0	0	35	0.90	0.89	0.91
Time constraint	10	50	18.5	11.4	17.5	19.0	0	46	0.91	0.90	0.92
Departmental influence	3	15	4.8	4.4	4.0	7.0	0	15	0.81	0.74	0.83
Professional identity	3	15	6.0	4.3	6.0	7.0	0	14	0.81	0.80	0.81
Student interaction	5	25	6.2	5.5	5.0	10.0	0	20	0.83	0.84	0.83

The correlation between the FSI score and participants’ perception of their depression, anxiety, and stress symptoms severity were also examined (Table 4). The FSI score was significantly correlated with participants’ perception of their depression (*P* < 0.001) and stress (< 0.001) symptoms severity.

**Table 4:** Pearson correlation (r) between Faculty Stress Index (FSI) score and depression, anxiety, and stress (*n* = 112)

		Depression	Anxiety	Stress
FSI	r	0.320	0.124	0.413
	*p*-value	< 0.001*	0.194	< 0.001*

*p < 0.05 indicating significant association

Results also showed that the mean score of FSI is 60.6 for those who reported at least moderate symptoms of depression (P = 0.005), and 67.0 for those who reported at least moderate symptoms of stress (P < 0.001) (Table 5). Under all the subscales of the faculty stress scores, participants were more likely to score high under at least ‘moderate’ (14+) symptoms of depression and at least ‘moderate’ (19+) symptoms of stress than normal/mild symptoms with the highest score related to the time constraint subscale; meanwhile, the lowest score is reported under the departmental influence.

**Table 5:** Depression, anxiety, and stress by Faculty Stress Index (FSI) and other selected participants characteristics

		Depression		Anxiety		Stress	
	Total	**At least ‘moderate’ (14+)**	p-value	**At least ‘moderate’ (10+)**	p-value	**At least ‘moderate’ (19+)**	p-value
N	112 (100.0%)	**33 (29.5%)**		**71 (63.4%)**		**29 (25.9%)**	
**Total FSI score, mean(SD)**	48.8 (29.4)	**60.6 (23.1)**	0.005	**49.1 (28.0)**	0.893	**67.0 (21.7)**	< 0.001
**FSI domains scores, n (%)**							
Reward and recognition	13.3 (9.3)	**16.6 (7.2)**	0.014	**12.8 (8.3)**	0.462	**17.7 (6.5)**	0.003
Time constraint	18.5 (11.4)	**22.3 (9.5)**	0.022	**18.8 (10.9)**	0.734	**25.9 (9.7)**	< 0.001
Departmental influence	4.8 (4.4)	**6.6 (4.0)**	0.004	**4.7 (4.1)**	0.632	**7.0 (3.7)**	0.002
Professional identity	6.0 (4.3)	**7.4 (3.5)**	0.020	**6.1 (4.0)**	0.609	**7.7 (3.5)**	0.012
Student interaction	6.2 (5.5)	**7.7 (6.1)**	0.067	**6.7 (5.6)**	0.219	**8.9 (5.6)**	0.002
**Age in years, n (%)** ^**^**^							
<39	36 (32.4%)	**15 (45.5%)**	0.080	**23 (32.4%)**	0.362	**11 (37.9%)**	0.353
40–49	36 (32.4%)	**11 (33.3%)**		**26 (36.6%)**		**11 (37.9%)**	
50+	39 (35.1%)	**7 (21.2%)**		**22 (31.0%)**		**7 (24.1%)**	
**Gender, n (%)**							
Female	52 (46.4%)	**20 (60.6%)**	0.052	**30 (42.3%)**	0.244	**18 (62.1%)**	0.050
Male	60 (53.6%)	**13 (39.4%)**		**41 (57.7%)**		**11 (37.9%)**	
**Nationality, n (%)**							
Non-Qatari	95 (84.8%)	**26 (78.8%)**	0.260^#^	**55 (77.5%)**	0.005	**21 (72.4%)**	0.039^#^
Qatari	17 (15.2%)	**7 (21.2%)**		**16 (22.5%)**		**8 (27.6%)**	
**College, n (%)**							
Other departments and centers	25 (22.3%)	6 (18.2%)	0.182	12 (16.9%)	< 0.001	4 (13.8%)	0.520
Arts and Science, Engineering	32 (28.6%)	14 (42.4%)		25 (35.2%)		10 (34.5%)	
Humanities	29 (25.9%)	8 (24.2%)		26 (36.6%)		9 (31.0%)	
Health-related	26 (23.2%)	5 (15.2%)		8 (11.3%)		6 (20.7%)	
**Highest academic degree, n (%)**							
BSc/ MSc	27 (24.1%)	**11 (33.3%)**	0.140	**17 (23.9%)**	0.958	**8 (27.6%)**	0.611
PhD	85 (75.9%)	**22 (66.7%)**		**54 (76.1%)**		**21 (72.4%)**	
**Administrative role, n (%)**							
No	64 (57.1%)	**19 (57.6%)**	0.952	**42 (59.2%)**	0.571	**15 (51.7%)**	0.493
Yes	48 (42.9%)	**14 (42.4%)**		**29 (40.8%)**		**14 (48.3%)**	
**Duration at QU in years, n (%)**							
<=2	31 (27.7%)	**9 (27.3%)**	0.110	**14 (19.7%)**	0.046	**4 (13.8%)**	0.027
>2 to 5	31 (27.7%)	**5 (15.2%)**		**22 (31.0%)**		**6 (20.7%)**	
5+	50 (44.6%)	**19 (57.6%)**		**35 (49.3%)**		**19 (65.5%)**	
**Employment type, n (%)**							
Full-time	103 (92.0%)	**27 (81.8%)**	0.019^#^	**64 (90.1%)**	0.350	**27 (93.1%)**	1.000^#^
Part-time	9 (8.0%)	**6 (18.2%)**		**7 (9.9%)**		**2 (6.9%)**	
**Sleep, at least 7 h per night, n (%)**							
No	55 (49.1%)	**20 (60.6%)**	0.116	**33 (46.5%)**	0.464	**18 (62.1%)**	0.105
Yes	57 (50.9%)	**13 (39.4%)**		**38 (53.5%)**		**11 (37.9%)**	
**Physical activity, at least 150 min per week, n (%)**							
No	58 (51.8%)	**19 (57.6%)**	0.428	**40 (56.3%)**	0.205	**19 (65.5%)**	0.086
Yes	54 (48.2%)	**14 (42.4%)**		**31 (43.7%)**		**10 (34.5%)**	
**Smoking, cigarettes/ vape/ nargileh, n (%)**							
No	103 (92.0%)	**28 (84.8%)**	0.121^#^	**64 (90.1%)**	0.482^#^	**25 (86.2%)**	0.232^#^
Yes	9 (8.0%)	**5 (15.2%)**		**7 (9.9%)**		**4 (13.8%)**	
**Medical diagnosis**^**&**^, **n (%)**							
No	85 (75.9%)	**25 (75.8%)**	0.983	**55 (77.5%)**	0.609	**20 (69.0%)**	0.311
Yes	27 (24.1%)	**8 (24.2%)**		**16 (22.5%)**		**9 (31.0%)**	
**Mental health diagnosis**^**&&**^, **n (%)**							
No	97 (86.6%)	**24 (72.7%)**	0.012^#^	**58 (81.7%)**	0.044	**22 (75.9%)**	0.061^#^
Yes	15 (13.4%)	**9 (27.3%)**		**13 (18.3%)**		**7 (24.1%)**	

p-values are from chi-square tests, ^#^Fisher exact tests, or two-sample ttest

^**^**^1 missing observation

^**&**^Includes hypertension, heart disease, diabetes, dyslipidemia, chronic kidney disease, chronic lung disease, or cancer

^**&&**^Includes schizophrenia, panic attacks, bipolar disorder, eating disorders, or others

In regards to participants’ demographics, female participants were more likely to score higher under at least ‘moderate’ symptoms of depression (60.6%) and stress (62.1%) compared to males (P = 0.052, 0.050, respectively) (Table 5). Non-Qatari participants scored higher under at least ‘moderate’ symptoms of anxiety (77.5%) and stress (72.4%), compared to Qatari participants (*P* = 0.005, 0.039, respectively),

;and participants from humanities (Law, Business, Sharia and Islamic Studies, and Education) (36.6%) were more likely to score higher under at least ‘moderate’ symptoms of anxiety compared to those from health-related colleges (11.3%) (P = < 0.001). Moreover, participants who had been for more than 5 years at the university scored higher under at least ‘moderate’ symptoms of anxiety (49.3%) and stress (65.5%) compared to those who spent less than 5 years at the university (P = 0.046, 0.027, respectively); meanwhile, full-time employees (96.2%) were more likely to report a higher score under at least ‘moderate’ symptoms of depression compared to part-time participants (P = 0.019). Finally, those who reported that they were not diagnosed with any mental health issue, were more likely to report at least ‘moderate’ symptoms of depression (72.7%)) and anxiety (81.7%) compared to those who reported being diagnosed with any mental health issue (*P* = 0.012, 0.044, respectively).

From the crude multivariable analyses (Table 6), the results showed that for every 10 points increase in FSI score, the prevalence of at least ‘moderate’ depression increased by 15% (P = 0.002) and of at least ‘moderate’ stress by 24% (P = < 0.001). All the subscales of the faculty stress scores were significantly associated with at least ‘moderate’ depression and stress symptoms severity. Under depression, it ranged between a Prevalence Ratio (PR) of 1.15 (95%CI 1.03, 1.29) for time constraint and 1.55 (95%CI 1.17, 2.06) for departmental influence related stressors. For example, for every 5-unit increase in departmental influence score, the prevalence of at least ‘moderate’ symptoms severity was higher by 55% compared to Normal/ Mild symptoms severity (P = 0.002). Under stress, it ranged between a PR of 1.29 (95%CI 1.11, 1.49) for reward and recognition and 1.67 (95%CI 1.24, 2.26) for departmental influence related stressors. For example, for every 5-unit increase in departmental influence score, the prevalence of at least ‘moderate’ symptoms severity was higher by 67% compared to Normal/ Mild symptoms severity (*P* = 0.001).

**Table 6:** Crude prevalence ratio (PR) for at least ‘moderate’ depression, anxiety, or stress

	Depression		Anxiety		Stress	
	PR [95% CI]	*p*-value	PR [95% CI]	*p*-value	PR [95% CI]	*p*-value
Total FSI score/10	1.15 [1.05,1.25]	0.002	1.00 [0.95,1.05]	0.896	1.24 [1.13,1.35]	< 0.001
Reward and recognition score/5	1.21 [1.05,1.39]	0.008	0.97 [0.90,1.05]	0.485	1.29 [1.11,1.49]	0.001
Time constraint score/5	1.15 [1.03,1.29]	0.013	1.01 [0.95,1.08]	0.742	1.33 [1.18,1.49]	< 0.001
Departmental influence score/5	1.55 [1.17,2.06]	0.002	0.96 [0.81,1.14]	0.645	1.67 [1.24,2.26]	0.001
Professional identity score/5	1.51 [1.09,2.09]	0.014	1.04 [0.88,1.24]	0.621	1.63 [1.14,2.33]	0.007
Student interaction score/5	1.26 [0.98,1.62]	0.069	1.08 [0.96,1.22]	0.206	1.50 [1.16,1.95]	0.002
**Age in years**						
<39	1.00		1.00		1.00	
40–49	0.73 [0.39,1.38]	0.334	1.13 [0.82,1.56]	0.452	1.00 [0.50,2.01]	1.000
50+	0.43 [0.20,0.94]	0.034	0.88 [0.61,1.28]	0.511	0.59 [0.25,1.36]	0.212
**Gender**						
Female	1.00		1.00		1.00	
Male	0.56 [0.31,1.02]	0.058	1.18 [0.89,1.58]	0.254	0.53 [0.28,1.02]	0.057
**Nationality**						
Non-Qatari	1.00		1.00		1.00	
Qatari	1.50 [0.78,2.91]	0.224	1.63 [1.32,2.00]	< 0.001	2.13 [1.13,4.01]	0.019
**College**						
Other departments & centers	1.00		1.00		1.00	
Arts and Science, Engineering	1.82 [0.82,4.07]	0.143	1.63 [1.04,2.55]	0.034	1.95 [0.69,5.52]	0.207
Humanities	1.15 [0.46,2.88]	0.766	1.87 [1.22,2.87]	0.004	1.94 [0.68,5.57]	0.218
Health-related	0.80 [0.28,2.31]	0.681	0.64 [0.32,1.30]	0.219	1.44 [0.46,4.53]	0.531
**Highest academic degree**						
BSc/ MSc	1.00		1.00		1.00	
PhD	0.64 [0.35,1.14]	0.127	1.01 [0.72,1.41]	0.958	0.83 [0.42,1.67]	0.607
**Administrative role**						
No	1.00		1.00		1.00	
Yes	0.98 [0.55,1.76]	0.953	0.92 [0.69,1.23]	0.577	1.24 [0.66,2.33]	0.495
**Duration at QU in years**						
<=2	1.00		1.00		1.00	
>2 to 5	0.56 [0.21,1.48]	0.239	1.57 [1.00,2.47]	0.049	1.50 [0.47,4.82]	0.496
5+	1.31 [0.68,2.53]	0.422	1.55 [1.01,2.38]	0.046	2.94 [1.10,7.89]	0.032
**Employment type**						
Full-time	1.00		1.00		1.00	
Part-time	2.54 [1.44,4.48]	0.001	1.25 [0.85,1.83]	0.249	0.85 [0.24,3.02]	0.799
**Sleep, at least 7 h per night**						
No	1.00		1.00		1.00	
Yes	0.63 [0.35,1.14]	0.124	1.11 [0.84,1.48]	0.468	0.59 [0.31,1.14]	0.114
**Physical activity, at least 150 min of physical activity**						
No	1.00		1.00		1.00	
Yes	0.79 [0.44,1.42]	0.433	0.83 [0.62,1.11]	0.213	0.57 [0.29,1.11]	0.097
**Smoke, cigarettes/vape/ nargileh (hookah)**						
No	1.00		1.00		1.00	
Yes	2.04 [1.05,3.98]	0.036	1.25 [0.85,1.83]	0.249	1.83 [0.81,4.12]	0.143
**Medical diagnosis** ^**&**^						
No	1.00		1.00		1.00	
Yes	1.01 [0.51,1.97]	0.983	0.92 [0.64,1.30]	0.624	1.42 [0.73,2.74]	0.301
**Mental health diagnosis** ^**&&**^						
No	1.00		1.00		1.00	
Yes	2.43 [1.41,4.17]	0.001	1.45 [1.12,1.88]	0.005	2.06 [1.07,3.97]	0.031

^**&**^Includes hypertension, heart disease, diabetes, dyslipidemia, chronic kidney disease, chronic lung disease, or cancer

^**&&**^Includes schizophrenia, panic attacks, bipolar disorder, eating disorders, or others

In regards to other sociodemographic characteristics, participants aged fifty years old and more were less likely by 57% (95%CI 0.20,0.94, P = 0.034) to report at least ‘moderate’ depression compared to younger participants, and male participants were less likely by 44% (95%CI 0.31,1.02, P = 0.058) and 47% (95%CI, 0.28,1.02, P = 0.057) to report at least ‘moderate’ depression, and at least ‘moderate’ stress, respectively, compared to females. On the other hand, Qatari faculty were 1.63 (95%CI 1.32,2.00, P = < 0.001) and 2.13 (95%CI 1.13,4.01, P = 0.019) times more likely to report at least ‘moderate’ anxiety and at least ‘moderate’ stress, respectively, compared to non-Qatari. Participants from humanities (Law, Business, Sharia and Islamic Studies, and Education), and “Arts and Sciences and Engineering” colleges were 1.87 (95%CI 1.22,2.87, P = 0.004) times and 1.63 (95%CI 1.04,2.55, P = 0.034) times more likely to report at least ‘moderate’ anxiety with compared to those from health-related colleges. Moreover, participants who had been working at the university between two-five years were 1.57 (95%CI 1.00,2.47, P = 0.049) times more likely to report at least ‘moderate’ anxiety symptoms than those who spent less time, and for those who had been working at the university for more than five years, in addition to report high under at least ‘moderate’ anxiety symptoms with PR 1.55 (95%CI 1.01,2.38, P = 0.046), they were 2.94 (95%CI 1.10,7.89, P = 0.032) times more likely to report at least ‘moderate’ stress symptoms compared to those who spent less that than five years at QU. In addition, part-time employees were 2.54 (95%CI 1.44,4.48, P = 0.001) times more likely to report at least ‘moderate’ depression symptoms compared to full-time employees. In regard to health and lifestyle factors, participants who used different tobacco products were 2.04 (95%CI 1.05,3.98, P = 0.036) times more likely to report high under at least ‘moderate’ depression symptoms compared to those who did not, and those who reported being diagnosed with mental health issues, were 2.43 (95%CI 1.41,4.17, *P* = 0.001), 1.45 (95%CI, 1.12,1.88, *P* = 0.005), and 2.06 (95%CI 1.07,3.97, *P* = 0.031) times more likely to score high under depression, anxiety, and stress, respectively, compared to those who were not diagnosed.

From the adjusted multivariable analyses (Table 7), the results showed that for every 10 points increase in FSI score, the prevalence of at least ‘moderate’ depression increased by 13% (P = 0.013) and the prevalence of at least ‘moderate’ stress by 30% (P < 0. 001). Qatari faculty were 1.61 (95%CI 1.16,2.25, P = < 0.004) times more likely to report at least ‘moderate’ anxiety and 2,38 (95%CI 2.38 [1.11,5.10], P = 0.026) times more likely to report at least ‘moderate’ stress compared to non-Qatari faculty. Moreover, participants from humanities were 1.72 (95%CI 1.07,2.79, P = 0. 027) times more likely to report at least ‘moderate’ anxiety compared to those from -health-related colleges.

**Table 7:** Adjusted prevalence ratio (PR) for at least ‘moderate’ depression, anxiety, or stress

	Depression	Depression	Anxiety	Anxiety	Stress	Stress
	PR [95% CI]	*p*-value	PR [95% CI]	*p*-value	PR [95% CI]	*p*-value
**Total FSI score/10**	1.13 [1.03,1.25]	0.013	1.03 [0.99,1.09]	0.174	1.30 [1.14,1.49]	< 0.001
**Age in years**						
<39	1.00		1.00		1.00	
40–49	0.71 [0.37,1.40]	0.327	1.20 [0.85,1.71]	0.296	0.66 [0.34,1.28]	0.216
50+	0.58 [0.23,1.47]	0.250	1.07 [0.70,1.65]	0.753	0.52 [0.23,1.18]	0.116
**Gender**						
Female	1.00		1.00		1.00	
Male	0.89 [0.48,1.64]	0.705	1.29 [0.93,1.80]	0.123	0.89 [0.44,1.78]	0.741
**Nationality**						
Non-Qatari	1.00		1.00		1.00	
Qatari	1.20 [0.64,2.25]	0.565	1.61 [1.16,2.25]	0.004	2.38 [1.11,5.10]	0.026
**College**						
Other departments & centers	1.00		1.00		1.00	
Arts and Science, Engineering	1.25 [0.47,3.28]	0.653	1.49 [0.86,2.56]	0.154	1.29 [0.36,4.59]	0.696
Humanities	1.12 [0.41,3.07]	0.822	1.72 [1.07,2.79]	0.027	2.03 [0.59,7.05]	0.263
Health-related	0.55 [0.17,1.83]	0.330	0.72 [0.37,1.40]	0.326	1.67 [0.44,6.33]	0.453
**Highest academic degree**						
BSc/ MSc	1.00		1.00		1.00	
PhD	1.09 [0.54,2.20]	0.811	0.77 [0.48,1.22]	0.262	0.79 [0.38,1.62]	0.517
**Administrative role**						
No	1.00		1.00		1.00	
Yes	0.79 [0.42,1.48]	0.466	0.84 [0.63,1.10]	0.203	0.72 [0.38,1.39]	0.329
**Duration at QU in years**						
<=2	1.00		1.00		1.00	
>2 to 5	0.84 [0.32,2.20]	0.723	1.58 [1.04,2.40]	0.030	2.51 [0.67,9.39]	0.170
5+	1.47 [0.60,3.57]	0.399	1.55 [0.98,2.46]	0.061	2.85 [0.88,9.16]	0.079
**Employment type**						
Full-time	1.00		1.00		1.00	
Part-time	2.53 [1.03,6.21]	0.043	1.33 [0.70,2.53]	0.389	1.01 [0.20,5.07]	0.991
**Sleep, at least 7 h per night**						
No	1.00		1.00		1.00	
Yes	0.68 [0.38,1.25]	0.216	1.14 [0.87,1.49]	0.330	0.85 [0.43,1.66]	0.634
**Physical activity, at least 150 min of physical activity**						
No	1.00		1.00		1.00	
Yes	0.98 [0.53,1.82]	0.950	0.90 [0.69,1.19]	0.461	0.59 [0.27,1.29]	0.188
**Smoke, cigarettes/ vape/ nargileh (hookah)**						
No	1.00		1.00		1.00	
Yes	1.75 [0.76,4.00]	0.186	0.86 [0.54,1.36]	0.515	2.39 [0.85,6.69]	0.098
**Medical diagnosis** ^**&**^						
No	1.00		1.00		1.00	
Yes	0.91 [0.43,1.91]	0.798	0.72 [0.49,1.04]	0.083	1.80 [0.89,3.63]	0.101
**Mental health diagnosis** ^**&&**^						
No	1.00		1.00		1.00	
Yes	2.05 [1.12,3.74]	0.019	1.89 [1.37,2.59]	< 0.001	0.87 [0.34,2.23]	0.767

^**&**^Includes hypertension, heart disease, diabetes, dyslipidemia, chronic kidney disease, chronic lung disease, or cancer

^**&&**^Includes schizophrenia, panic attacks, bipolar disorder, eating disorders, or others

The results also showed that participants who had spent between two- five years at the university were 1.58 (95%CI 1.04,2.40, P = 0.030) times more likely to report at least ‘moderate’ anxiety symptoms compared to those who spent less time and part-time employees were 2.53 (95%CI 1.03,6.21,, P = 0.043) times more likely to report at least ‘moderate’ depressions symptoms compared to full-time employees. Related to participants’ health status, those who had been diagnosed with mental health issues were 2.05 (95%CI 1.12,3.74, P = 0.019), and 1.89 (95%CI 1.37,2.59, P = 0.001) times more likely to report at least ‘moderate’ depression and anxiety symptoms, respectively, compared to those who were not diagnosed.

## Discussion

This study is the first in Qatar to gain insights into mental health and associated occupational stressors among faculty. While promoting human values and scholarly excellence, the ecosystem of higher education institutions is not completely free from adversity, and can put academics at risk of different mental health–related concerns [43]. The cumulative effects of increasing workloads, long working hours, and challenges with work–life balance have been described as the roots of occupational stress in academia [44], underscoring the need for a focused investigation on these concerns in our institution. This becomes especially imperative in light of both, national contexts of prioritizing mental health [45], and calls for scrutinizing and improving mental health in academia [46].

Upon assessment of self-perceived faculty mental health using DASS-21, it was intriguing to find a minimum of 30%, 63%, and 26% of the participants having at least moderate levels of depression, anxiety, and stress respectively. These figures exceed reported results in a study from 10 big universities in the US, with ranges between 6 and 26% [28]. The reported prevalence of anxiety also exceeds that reported in a systematic review and meta-analysis of mental health of the general population during COVID-19, with an estimate of 38% [47]. Nevertheless, our numbers are still lower than those previously reported among academics in Australia [48] and the United Kingdom [49], with findings of almost 50% risk of psychological illnesses. As such, directed interventions aiming at improving the well-being of academic staff and contributing to more conversations on this topic are ultimately needed. For example, Recently, Lim and Colleagues [50] found that DASS-21 scores showed an improvement in faculty after mental health training interventions and professional support. Likewise, a Spanish study found that multiple mental and physical approaches improved self-perceived stress among academics [51]. For anxiety, mostly self-perceived among our participants, examples of digital and web-based interventions have proved previously effective in academic settings [52], and may be tempting to investigate for faculty.

Exploring occupational stressors of faculty using FSI showed a mean FSI score of 48.8, in stark difference with higher values reported using the same measure in other studies from that tackled public [53] and private institutions [35]. Further, multiple factors contribute to faculty stress, as shown by the FSI domains. The time constraint domain scored highest among all five domains in terms of perceptions of stress by faculty, followed by the rewards and recognition domain. Regarding time constraints domain, this construct is related to stress from time management of various commitments including heavy workload, committee services, meetings, out-of-office duties, and social obligations, among others. The issue of faculty time restraints and extra working hours has been previously documented in literature [54, 55], with some reports of 40% of faculty working at least 10 extra hours per week [56]. As such, it is comprehensible that our participants perceived time demands as the main contributor to their workplace stress, in parallel to observations in academic institutions elsewhere. In modern universities with teaching, research and service missions, the production of knowledge through research, and the transmission of knowledge to students through teaching and to societal stakeholders through service, all have brought about an operational complexity, the sophistication of which is cascaded to faculty, pressuring them to accomplish more within a shorter time [57]. While faculty still consider themselves independent professionals, their traditional self-determination and autonomy regarding their working times have become subject to increasing scrutiny under the burden of calls for improved productivity, efficiency and accountability, thereby increasing occupational stress [58].

In terms of rewards and recognition, which was the second scoring domain in the FSI, participating faculty perceived evaluation criteria, community service recognition, teaching recognition, and other factors as drivers behind their stress. Previously, reward and recognition were shown to have significant correlation with different dimensions of work motivation and satisfaction in employees of various types of organizations not limited to academia, as reported by Danish and Colleagues [59]. Furthermore, rewards and recognition are regarded as top priorities for faculty motivation and/or satisfaction [60], as well as higher job performance [61]. More specifically, an investigation in an Australian university revealed that reward and recognition were perceived as actual barriers to promotion of faculty who did not conform to a ‘traditional’ structure of research expectations, whereby disadvantaged faculty from practice or professional backgrounds, or those who had heavy administrative roles, are not properly rewarded [62]. As such, our results conform with the body of evidence reporting the pressure that rewards and recognition exert on of faculty [63, 64]. This probably calls for motivation of faculty through proper recognition and appreciation, whereby flexible guidelines, discipline-specific performance expectations, and career development pathways are reconsidered. A holistic approach to rewarding a broad range of educational roles may be beneficial, and requires strong advocacy to create changes in academic rewards in the interest of better faculty motivation and well-being.

On another end, the perceived stress by faculty due to each of student interaction and professional identity domains received almost one third of the scores for time constraint and about half of the scores for reward and recognition domain. Furthermore, the departmental interaction domain perceived stress scored the least among the five FSI domains, and observations on these three domains deviate from other published data [35]. For instance, student interaction was perceived to cause highest stress levels among faculty in KSA, according to the findings of Iqbal and Colleagues [31]. An in-depth look into items of the student interaction domain reveals that it addresses faculty normal tasks with students, like student evaluation, preparing class presentations, being evaluated by students for performance, and advising students, even those who may be inadequately prepared. With the majority of our sample being full-time PhD holders above the age of 40, it is likely that most of them have a rich teaching record and an extensive experience with handling student-related matters, reducing the contribution of these matters to perceived occupational stress. For junior faculty, it has been reported that teaching tasks not only occupy much of their time allowance, but also requires reasonable efforts in dealing with interaction-based activities that are entirely different from analysis-based research, easily leading to mental overload [65]. This might not be the case for our population, mostly consisting of senior academics. However, our findings may highlight the call for investing in more interesting teaching activities, as this may nurture the pedagogical process, while also contributing to less stress among faculty. Likewise, professional identity, focused on research support and professional conferences attendance, ranked fourth on the domains of perceived stress, probably due to the university prioritizing research and encouraging faculty visibility by presenting their scholarly work externally. The least perception of occupational stress by faculty was in the departmental influence domain, a construct emphasizing on departmental evaluation and resolving department conflicts. The relatively favorable results in such domain may indicate that faculty have feelings of belonging, and that one’s contribution to the department is recognized and valued, perhaps contributing to less stressful work days. Faculty members who perceive less stress in this area may be more collegial and generate amiability to improve the working atmosphere of their department and institution [33].

In multivariable analyses, increase in FSI score was associated with statistically significant likelihood of increase in severity of both depression and stress. Hence, while faculty juggle their various responsibilities, trying to sufficiently manage their time, get rewarded for achievements, and attend on various student needs, professional profiles, and departmental requirements, they may fall short of securing their own well-being, and can become at higher risk of encountering more severe mental conditions. Previously, Melnyk and Colleagues [28, 66] reported that healthy lifestyle, sleep, and physical activity were associated with lower severity of mental conditions among faculty, namely depression and anxiety. Moreover, a study showed that faculty with initially high levels of occupational stress had significant improvements in this condition after targeted stress management interventions [67]. Similarly, mindfulness programs [68], de-stressors like yoga and art therapy [69], and behavioral coaching interventions [70] have been reported to positively affect mental health of faculty. However, despite importance, all these attempts remain deficient in addressing specific and tailored demands of faculty working environments, and creating custom-made interventions targeting faculty stress attributes, like those explored by the various FSI domains. According to a systematic review on mental health of academics, minimal research on managing mental health among faculty exists, and only limited information that measures the outcome of various mental well-being strategies is available [71]. As such, further research and robust study designs are needed in this area to concentrate on faculty-specific stressors and how they can be ameliorated in the workplace. Establishing routine mental health assessment, effective communication strategies, and continuous support are all imminent to improve the mental well-being of academics.

Noteworthy, multivariable analysis also showed a significance of less likely depression levels of at least moderate severity among faculty aged 50 years and above. Additionally, faculty who were nationals were statistically more likely to report at least moderate anxiety and stress levels. The latter two findings are in parallel with those formerly reported by Ganji and Colleagues [72]. While it could be hypothesized that with age, individuals experience a growth in maturity, enabling them to cultivate resilience by navigating through diverse stressors over the years, leading to improved emotional regulation and a reduction in symptoms of depression [73, 74], the second finding of at least moderate anxiety and stress being more likely in Qatari faculty cannot be directly interpreted from our results, given that they constitute only 15% of the surveyed sample. Furthermore, faculty from humanities domains were more likely to report at least moderate anxiety compared to faculty from health-related colleges. In general, faculty and staff in medical schools may be inherently exposed to mental health issues among students, such issues being common among this population [75, 76], triggered by demanding medical curricula and high financial costs [77]. Constantinou and Colleagues [78], in their review of medical faculty, point out that those faculty acknowledge the importance of mental illness, discuss symptoms with their students and provide support, and embrace the idea of being trained in this field. As such, we anticipate that health faculty, due to their background, might have better awareness about mental health issues, possibly making them personally less perceiving of some of them, like anxiety. Moreover, given the nature of their profession, health faculty may have a higher level of empathy and understanding of mental health [79]. They also often work under high-stress environments [80], and are part of a sector that recognizes mental health significance [81]. It is possible that all these integral constructs in health faculty roles could make them less likely to report self-perceived anxiety, and this may be interesting for an additional, focused investigation. Likewise, the observation of higher likelihood of at least moderate anxiety levels in faculty who have been at QU for 2–5 years compared to those who spent less time, may indicate a probable timeframe during which faculty may become deeply engulfed in their various academic duties and during which proper self-care and external support to avoid anxiety may be needed. Also, the higher likelihood of at least moderate depression among part-time faculty is a result that warrants additional study, especially with the latter finding recently reported among part-time workers [82]. The stress of having different jobs and the worrisome feelings about job instability, may instigate more mental health issues among this group of faculty.

The strength of this study lies in being pioneer in addressing faculty mental health from our institution, using validated tools, and in the use of a bilingual survey design that captures the prevailing cultural diversity of the studied population. Moreover, our study establishes links between specific occupational stressors for faculty and their DASS-21 scores, laying the ground for job-specific mental health investigations. However, our study does have limitations. First, we cannot neglect recall bias in a self-administered instrument; second, we expect some participants to have dropped out while answering the survey given the length of the instrument and the multiple statements in both the FSI and DASS-21, causing loss of some responses. Also, better conclusions from this study would be drawn out if the FSI Arabic version was back-translated, to ensure it captures more explicitly the insights of faculty who answered it in Arabic. While giving a preliminary outlook on how the mental health of faculty can be portrayed, and what essential strains in the work environment are significantly implicated, more remains to be captured in such and similar inquiries. This includes structured, periodic assessment of mental health and well-being of faculty, and exploring the efficacy of interventions that aim at reducing their specific occupational stressors. The preliminary findings from this study could be seen from the lens of proper practical recommendations that can support faculty mental health. The recognition and awareness regarding the need to improve faculty mental health can be the first step for implementing measures that favor their well-being. Organizational level measures, fair allocation of workload, and time management training could pave the way towards better mental health for academic faculty and foster a supportive environment for their wellness and ability to successfully thrive throughout the academic landscape.

## Conclusion

In conclusion, perceived depression, anxiety, and stress in the academic setting is common among faculty, and mostly culminating from time constraints and faculty recognition to an extent higher than other factors like student interaction, professional identity and departmental issues. The higher participants perceived stress from their academic career, the more likely they were to experience more severe mental health symptoms, namely depression and stress. The implications of these findings indicate that controlling occupational stressors for faculty would be essential to avoid mental health conditions or at least, reduce their severity. Examining mental health conditions and their determinants for QU faculty members and purposeful consideration of the outcomes, will support the efforts of QU as a Healthy University, and will complement and guide its strategic efforts for a healthy campus. The results of this research will provide baseline evidence on the need for effective interventions towards occupational stress, orientation towards mental health, and informing policies on campus.

### Electronic supplementary material

Below is the link to the electronic supplementary material.

Supplementary Material 1

## Data Availability

The datasets generated and/or analyzed during the current study are not publicly available due to the ethical concerns and participant anonymity, but are available from the corresponding authors upon reasonable requests.
