# Supplementary material for: Associations between perceived occupational stressors and symptoms severity of depression, anxiety and stress among academic faculty: First cross-sectional study from Qatar
Source: BMC Psychol. 2024 May 28;12:302. doi: 10.1186/s40359-024-01801-x (PMC11134782; doi:10.1186/s40359-024-01801-x)
Supplement: Supplementary file 1 — Supplementary Material 1 [file 40359_2024_1801_MOESM1_ESM.docx]

**Supplementary file 1: English version of the survey used in this study**

Dear Participant,

We are researchers from the Academic Quality Department, QU Health, at Qatar University, and we are conducting a research study that aims to explore mental health and well-being among faculty members at Qatar University and investigate factors associated with depression, anxiety, and stress.

This study is approved by the Qatar University Institutional Review Board (QU-IRB) with the approval number:  QU-IRB 1900-E/23. If you have any questions related to ethical compliance of the study, you may contact them at QU-IRB@qu.edu.qa or at 4403 5307. QU-IRB and Ministry of Public Health (MoPH) can access the data collected in this survey (if needed).

You have received our email to participate in this study because you are a current faculty member at Qatar University.  If you decide to participate, you will be presented with a brief survey with questions about your health behavior and mental health. You will require approximately 10 minutes to complete our survey.

Participation in this study will allow us to gain information about the prevalence of stress, anxiety, depression, and burnout among QU faculty; to analyze levels of resilience, and to correlate such observations to various factors related to workload, college, and others.  The outcomes of this project shall reveal baseline data about mental health and well-being among QU faculty, thus helping to establish, promote, and support evidence-based wellness programming that improves the healthy behaviors of faculty, and results in less stress and burnout in the workplace.

**Voluntary Participation and Withdrawal:** Your participation in this research is voluntary, and you may decide to withdraw at any time. Skipping survey questions or deciding to withdraw will not will not result in any penalty or loss of benefits to which you would otherwise be entitled.

**Confidentiality:**Data will be collected anonymously, and will be saved on the computer of the PI where they are password protected; only the PI and Co-I to have access to the data. Data will be treated anonymously. No name or any data related to subjects’ identity will be used for data analysis or publication.

If you are willing to participate in this research, please click the “I am a current faculty member at QU and consent to participate in this study” button below. By clicking this, you acknowledge that your participation in the study is voluntary, you are a faculty member QU, and you are aware that you may choose to terminate your participation in the study at any time and for any reason. Please note that this survey will be best displayed on a laptop or desktop computer. Some features may be less compatible for use on a mobile device.

Kindly contact the PI, Dr. Dalal Hammoudi, (email: dhammoude@qu.edu.qa, office phone: 4403-6087) in case you have questions, concerns, or complaints about this study.

**Please select one of the following options:**

- I am a current faculty member at QU and consent to participate in this study.
- I do not consent; I do not wish to participate.

Section 1: Demographic data

1. Age (open-ended)
2. Gender:

- Male
- Female

1. Nationality:

- Qatari
- Other nationalities

1. College:

- Medicine
- Dental medicine
- Pharmacy
- Health sciences
- Nursing
- Education
- Engineering
- Arts and Science
- Law
- Business and Economics
- Sharia and Islamic Studies

1. Highest academic degree:

- PhD
- Masters
- Bachelor

1. Rank:

- Professor
- Associate professor
- Assistant professor
- Lecturer
- Teaching assistant

1. Employment type:

- Full-time
- Part-time

1. Administrative role:

- Yes
- No

Section 2: Health and lifestyle

1. Do you sleep at least 7 hours per night?

- Yes
- No

1. Do you perform at least 150 minutes of physical activity per day?

- Yes
- No

1. Do you smoke cigarettes/vape?

- Yes
- No

1. Are you diagnosed with any medical condition like hypertension, heart disease, diabetes, dyslipidemia, chronic kidney disease, chronic lung disease, or cancer?

- Yes
- No

1. Are you diagnosed with a mental health disorder like anxiety, depression, schizophrenia, panic attacks, bipolar disorder, eating disorders, or others?

- Yes
- No

1. Do you take any medications for mental health conditions like tranquillizers, antidepressants, antipsychotics, mood stabilizers, sleeping pills, or lithium?

- Yes
- No

Section 3: The Faculty Stress Index (FSI)

1. Rewards and recognition subscale

Please rate the following statements from Not Applicable Pressure (0), Very Slight Pressure (1), Slight Pressure (2), Moderate Pressure (3), Some Pressure (4), Excessive Pressure (5) regarding the factors that derive stress from inadequate rewards, insufficient recognition, and unclear expectations in teaching, research, and service.

1. Receiving inadequate university recognitions for community services
2. Having insufficient reward for institutional / departmental services
3. Receiving insufficient recognition for teaching performance
4. Not having clear criteria for evaluating service activities
5. Lacking congruency in institutional, departmental and personal goals
6. Receiving insufficient institutional recognition for research performance
7. Not having clear criteria for evaluation of research and publication activities
8. Time constraint subscale

Please rate the following statements from Not Applicable Pressure (0), Very Slight Pressure (1), Slight Pressure (2), Moderate Pressure (3), Some Pressure (4), Excessive Pressure (5), regarding your feelings of insufficient time to keep abreast of current development, inadequate time for class preparation, interruptions from telephones, writing memos, attending meetings, too heavy workload, and job demands interfering with your personal activities.

- 1. Having insufficient time to keep abreast of current developments in my field
  2. Assignments of duties that take me away from my office
  3. Being interrupted frequently by telephone calls and drop-in visitors
  4. Having inadequate time for teaching preparation
  5. Writing letters and memos and responding to other paperwork
  6. Feeling that I have too heavy workload, one that I cannot possibly finish during normal work day
  7. Attending meetings which take up too much time
  8. Participating in work-related activities outside regular working hours
  9. Meeting social obligations (parties, volunteer work) expected of me because of my position
  10. Having job demands which interfere with other personal activities (recreation, family and other interest)

1. Departmental influence subscale

Please rate the following statements from Not Applicable Pressure (0), Very Slight Pressure (1), Slight Pressure (2), Moderate Pressure (3), Some Pressure (4), Excessive Pressure (5), regarding the factors that deal with influence of chairs’ decisions, resolving differences, and impact on departmental and institutional decision-making.

1. Resolving differences with my chair
2. Lacking personal impact on departmental/institutional decision making
3. Not knowing how my chair evaluates my performance
4. Professional identity subscale

Please rate the following statements from Not Applicable Pressure (0), Very Slight Pressure (1), Slight Pressure (2), Moderate Pressure (3), Some Pressure (4), Excessive Pressure (5), regarding the factors that deal with your reputation, which is built on scholarship: publications, presentations in conferences, grants, and research.

1. Making presentations at professional conferences and meetings
2. Imposing excessively high self-expectations
3. Securing financial support for my research
4. Student interaction subscale

Please rate the following statements from Not Applicable Pressure (0), Very Slight Pressure (1), Slight Pressure (2), Moderate Pressure (3), Some Pressure (4), Excessive Pressure (5), regarding your interaction with students.

1. Evaluating the performance of students
2. Having students evaluate my teaching performance
3. Teaching/advising inadequately prepared students
4. Resolving differences with students
5. Making class presentations

Section 4: The Depression, Anxiety, Stress Scale-21 items (DASS-21)

Please read each statement and select a number 0, 1, 2 or 3 which indicates how much the statement applied to you over the past week. There are no right or wrong answers. Do not spend too much time on any statement.

PLEASE RATE AS FOLLOWS:

0 Did not apply to me at all - NEVER
1 Applied to me to some degree, or some of the time - SOMETIMES
2 Applied to me to a considerable degree, or a good part of time - OFTEN
3 Applied to me very much, or most of the time - ALMOST ALWAYS

1. I found it hard to wind down.
2. I was aware of dryness of my mouth.
3. I couldn't seem to experience any positive feeling at all.
4. I experienced breathing difficulty (eg, excessively rapid breathing, breathlessness in the absence of physical exertion).
5. I found it difficult to work up the initiative to do things.
6. I tended to over-react to situations.
7. I experienced trembling (eg, in the hands).
8. I felt that I was using a lot of nervous energy.
9. I was worried about situations in which I might panic and make a fool of myself.
10. I felt that I had nothing to look forward to.
11. I found myself getting agitated.
12. I found it difficult to relax.
13. I felt down-hearted and blue.
14. I was intolerant of anything that kept me from getting on with what I was doing.
15. I felt I was close to panic.
16. I was unable to become enthusiastic about anything.
17. I felt I wasn't worth much as a person.
18. I felt that I was rather touchy.
19. I was aware of the action of my heart in the absence of physical exertion (eg, sense of heart rate increase, heart missing a beat).
20. I felt scared without any good reason.
21. I felt that life was meaningless.

**Thank you for completing this survey.**
